# TNF-α-1031T/C gene polymorphism as a predictor of malnutrition in patients with gastric cancer

**DOI:** 10.3389/fnut.2023.1208375

**Published:** 2023-07-18

**Authors:** Liang Fu, Changzhen Lei, Yingxun Chen, Ruiyun Zhu, Minling Zhuang, Liping Dong, Xianghong Ye, Lushan Zheng, Daojun Gong

**Affiliations:** ^1^Department of Nursing, Affiliated Jinhua Hospital, Zhejiang University School of Medicine, Jinhua, Zhejiang, China; ^2^Central Laboratory, Affiliated Jinhua Hospital, Zhejiang University School of Medicine, Jinhua, Zhejiang, China; ^3^Department of Gastrointestinal Surgery, Affiliated Jinhua Hospital, Zhejiang University School of Medicine, Jinhua, Zhejiang, China

**Keywords:** gastric cancer, malnutrition, TNF-α, gene polymorphism, single nucleotide polymorphisms

## Abstract

**Introduction:**

Malnutrition is a complex clinical syndrome, the exact mechanism of which is yet not fully understood. Studies have found that malnutrition is associated with anorexia and inadequate intake, tumor depletion, leptin, tumor-induced metabolic abnormalities in the body, and catabolic factors produced by the tumor in the circulation and cytokines produced by the host immune system. Among these, single nucleotide polymorphisms (SNPs) are present in the gene encoding the pro-inflammatory cytokine TNF-α.

**Aim:**

The objective of this study was to investigate TNF-α -1,031 T/C gene polymorphism as an unfavorable predictor of malnutrition in patients with gastric cancer.

**Methods:**

The study group consisted of 220 gastric cancer patients treated at Affiliated Jinhua Hospital, Zhejiang University School of Medicine. Malnutrition was mainly assessed by the Global Consensus on Malnutrition Diagnostic Criteria (GLIM). DNA was extracted from peripheral leukocytes of whole blood samples using an animal DNA extraction kit. DNA was amplified using a 1.1× T3 Super PCR mixture and genotyped using SNP1 software.

**Results:**

There are three major genetic polymorphisms in TNF-α. Among the 220 patients with gastric cancer, there were 7 patients with the CC genotype, 61 with the CT genotype and 152 with the TT genotype. Compared to patients with the TT genotype, patients with the C allele had an approximately 2.5-fold higher risk of developing malnutrition (*p* = 0.003; OR = 0.406). On the basis of multivariate analysis, patients with the CC genotype had an approximately 20.1-fold higher risk of developing malnutrition (*p* = 0.013; OR = 20.114), while those with the CT genotype had an almost 3.7-fold higher risk of malnutrition (*p* = 0.002; OR = 3.218).

**Conclusion:**

SNP (−1,031 T/C) of the TNF-α may be a useful marker in the assessment of the risk of nutritional deficiencies in gastric cancer patients. Patients with gastric cancer carrying the C allele should be supported by early nutritional intervention, but more research is still needed to explore confirmation.

## 1. Introduction

Gastric cancer (GC) is the fifth most common malignancy worldwide and one of the three leading causes of tumor-related deaths (1). Despite the great progress in early diagnosis and treatment in the past decades, the overall survival rate of gastric cancer patients remains low (2, 3). Malnutrition is extremely common among gastric cancer patients due to chronic tumor depletion, preoperative neoadjuvant chemotherapy, and inadequate nutritional intake due to pyloric obstruction. It is estimated that the malnutrition rate can be as high as 50% in stage II and III gastric cancer patients 12 months after gastrectomy, which not only affects the outcome of treatment and postoperative complications but is also significantly associated with poor prognosis (4–7). The adverse effects of malnutrition in the perioperative period have become a growing concern among clinicians. Therefore, nutritional assessment has become an important part of the clinical management of oncology patients and is the main basis for determining whether to initiate nutritional interventions in a timely manner.

Malnutrition is a complex clinical syndrome that can occur in a variety of chronic or end-stage diseases, including malignancies, parasitic infections, and chronic heart failure (8, 9). Its main manifestations are anorexia, weakness, wasting, disturbances in glucose metabolism, lipolysis, muscle atrophy, and hypoproteinaemia. The specific manifestation is the progressive depletion of the body’s reserve adipose tissue and skeletal muscle (10). From a nutritional point of view, the factors associated with this condition are thought to be anorexia and increased energy expenditure. However, as nutritional supplementation alone cannot improve malnutrition, anorexia alone cannot explain the complex metabolic changes that occur during malnutrition. This suggests that undernutrition is not the underlying cause of malnutrition and that more complex metabolic mechanisms exist. Progressive loss of muscle mass is a common feature of sarcopenia and malnutrition, leading to major functional impairment. The high rate of muscle catabolism is thought to be due to excessive activation of calcium-dependent and ATP-ubiquitin-dependent proteolytic pathways (11, 12). However, malnutrition refers to an imbalance between energy intake, energy expenditure and quality of nutrient intake and (13, 14), like cachexia, it may be associated with diseases with inflammatory activity (14), whereas sarcopenia is a progressive, generalized skeletal muscle disease that may or may not be associated with other diseases or inflammatory processes (15). Nevertheless, like malnutrition and cachexia, loss of muscle mass is one of the primary features of sarcopenia. In addition, malnutrition is a strong predictor of sarcopenia and myosteatosis (16). Therefore, only if all these clinical concepts are fully clarified at the time of assessment will it be possible to achieve appropriate preventive and therapeutic measures, especially when it comes to the oncological process. Currently, tools commonly used in clinical practice to screen and assess patients for malnutrition include the Nutrition Risk Screening 2002 (NRS-2002), the Malnutrition Universal Screening Tool (MUST), and the Patient Generated Subjective Global Assessment (PG-SGA) (17, 18), but there is still a lack of a globally accepted diagnostic basis for malnutrition (19). In this context, a new diagnostic criterion for malnutrition, the Global Consensus on Malnutrition Diagnostic Criteria (GLIM) (14), was proposed.

Malnutrition has the highest prevalence in malignancies and is a serious manifestation of advanced stages in patients with malignancies. The causes of cancer malnutrition are not fully understood, and the role of cancer-inducing inflammatory cytokines such as IL⁃6 and TNF⁃α in its occurrence and development is gradually being recognized (20, 21). Meanwhile, an increasing number of studies have confirmed that cytokine levels in the human organism are regulated by host genes and that cytokine gene polymorphic sequences may be genetic markers of susceptibility or clinical manifestations of certain infectious diseases (22). As SNPs do not change during the disease process, in addition, the risk of malnutrition is individual and does not appear to be related to demographic or most clinical factors. Therefore, it has been proposed that the risk of malnutrition may be related to genetic predisposition (23, 24). TNF-α is probably the most characteristic cytokine in malnutrition. Up until now, several functional SNPs within the TNF-α gene have been identified and described as cancer-related genetic alterations (25). Under normal conditions, TNF-α has protective effects against tumors and infections, but persistent high expression or dysregulation of its relationship with other cytokines can cause fever, shock, and malnutrition in the body. TNF-α binds to TNF receptors on the surface of muscle cells and induces the expression of proteins such as MURF-1 and MAFbx through the NF-κB signaling pathway, activating the ubiquitin-proteasome pathway (UPP) to degrade muscle proteins (26, 27). TNF-α also promotes lipolysis by activating the MAPK (p44/42) and Jun N-terminal kinase pathways (28). In addition to lipid and muscle wasting, elevated TNF-α can lead to anorexia, activate skeletal muscle protein degradation, inhibit protein synthesis, induce insulin resistance and mediate systemic inflammation in cancer malnutrition, further exacerbating malnutrition (29). Several polymorphisms within the TNF-α promoter region (-238, -244, -308, -376, -489, -575, -610, -851, -857, -863, -1031) have been reported to affect the expression of TNF-α genes and disease susceptibility (23, 25, 30–36). The most recent of these studies demonstrated that the TNF-α-1031T/C SNP (rs1799964) may be associated with malnutrition (25, 33). Therefore, the aim of this study was to assess the TNF-α gene polymorphism (−1,031 T/C, rs1799964) as a predictor of malnutrition or prognostic factors in patients with gastric cancer.

## 2. Materials and methods

### 2.1. Study group

The study group consisted of 220 patients with gastric cancer attending Affiliated Jinhua Hospital, Zhejiang University School of Medicine between December 2020 and February 2022. Inclusion criteria: (1) age 18 years or older; (2) diagnosis of gastric cancer confirmed by clinicopathology; (3) no anti-tumor treatment has been received for this admission; (4) informed consent to the study. Exclusion criteria: (1) presence of cognitive impairment, psychiatric disease, or other conditions that make participation in the study inappropriate; (2) very poor prognosis, with an expected survival of fewer than 12 weeks for patients; (3) all diseases with a background of chronic inflammatory pathology, such as diabetes, obesity, ischaemic heart disease, chronic kidney disease, autoimmune diseases.

The study was approved by the Medical Ethics Committee of Jinhua Municipal Central Hospital [(Research) 2020-Ethical Review-240, (Research) 2021-Ethical Review-16 and (Research) 2021-Ethical Review-16]. All patients in the study signed an informed consent form.

### 2.2. General information

Patients’ gender, age, height, weight, time of diagnosis, tumor stage and treatment modality are collected through the electronic medical record system. Patients with gastric cancer are classified into “upper 1/3″, “middle 1/3″ and “lower 1/3″ according to the primary location of the tumor. The TNM staging was performed according to the guidelines of the International Union Against Cancer (UICC). The following information was also collected: weight loss, smoking, and drinking habits, with patients divided into “current,” “ever” and “never” groups according to their smoking habits. The patient’s performance status was assessed according to the Eastern Cooperative Oncology Group (ECOG) scale. Alcohol consumption was assessed according to the International Statistical Classification of Diseases and Related Health Problems (ICD).

### 2.3. Measurements

As the only malnutrition assessment tool currently developed through global consensus, the Global Consensus on Malnutrition Diagnostic Criteria (GLIM) (14) was selected as the primary measurement tool in this study. The Nutritional Risk Screening 2002 (NRS-2002) scale (37) and the Patient Generated Subjective Global Assessment (PG-SGA) (38) are now routinely used in our hospitals to assess malnutrition. Thus, patients’ nutritional status was assessed according to these three tools. The NRS-2002 assesses the risk of malnutrition based on the patient’s nutritional status, severity of disease, and age. According to the NRS-2002, an NRS score of <3 is considered a low risk of malnutrition and a score ≥ 3 is considered a high risk of malnutrition (37). The GLIM is consisting of three phenotypic criteria (non-autonomic body mass loss, low body mass index, and muscle loss) and two etiological criteria (reduced intake or impaired digestion and absorption, inflammation or disease burden) (14, 39). A diagnosis of malnutrition should have at least 1 phenotypic criterion and 1 etiological criterion. Patients were diagnosed as malnourished or not according to GLIM, and were divided into two groups: the malnutrition group and the non-malnutrition group. PG-SGA is a quick, valid and reliable nutrition assessment tool that enables malnourished hospital patients with cancer to be identified and triaged for nutrition support. According to the PG-SGA scale, patients are classified as malnourished when scoring four and above (38).

### 2.4. Genotyping

DNA was isolated from whole blood samples using the Animal DNA Extraction Kit (TSINGK, China). DNA was amplified using 1.1 x T3 Super PCR mixture (TSINGK, Beijing, China) followed by electrophoresis and Sanger sequencing. And genotyping was detected using the SNP1 software (TSINGK, China). Each genotyping step followed the protocol conditions provided by the manufacturer.

### 2.5. Statistical analysis

Statistical analyses were performed using SPSS 26.0 software. The distribution of demographic and nutritional factors in patients with different genotypes of the TNF-α gene was tested using Fisher’s exact test and Chi-squared test, and their odds ratio (OR) and 95% confidence intervals (CI) were calculated, with *p* < 0.05 considered statistically significant. As the continuous data had a non-normal distribution, the median and range were used as measures of their concentration and dispersion. Logistic regression models were used in the risk of nutritional disorders with statistically significant factors from univariate analysis (*p* < 0.05) as included variables.

## 3. Results

### 3.1. General characteristics of the study group

The study included 220 patients with GC. According to the TNM classification, 51.82% of the patients were in stage III and 11.82% in stage IV. The median age of the patients was 65 years (range: 33–90 years). The median weight of patients was 55.0 kg (range: 38.0–95.0 kg) and the median BMI was 20.4 kg/m2 (range: 14.2–32.8 kg/m2). There was a male predominance (70.00%). The pathology of the majority of patients was adenocarcinoma (81.82%). 57.73% of patients had GC located in the lower third of the stomach. 68.64% of patients underwent surgery for gastric cancer, with 48.34% of them undergoing total gastrectomy. The malnutrition prevalence was 45.0% according to GLIM. The number of patients with malnutrition was 142 (64.55%) assessed by the PG-SGA. Of the 220 patients with gastric cancer, 7 were of the CC genotype, 61 of the CT genotype and 152 of the TT genotype (Table 1).

**Table 1:** Characteristics of the study group.

Variable		Study group (*n* = 220)
Gender	Male	154(70.00%)
	Female	66(30.00%)
Age [years]	Median (range)	65(33–90)
	≥65	114(51.82%)
	<65	106(48.18%)
Histopathological diagnosis	Adenocarcinoma	180(81.82%)
	Non-adenocarcinoma	40(18.18%)
Tumor location	Upper third	43(19.54%)
	Middle third	50(22.73%)
	Low third	127(57.73%)
T stage	T1	25(11.36%)
	T2	32(14.55%)
	T3	79(35.91%)
	T4	84(38.18%)
N stage	N0	46(20.91%)
	N1	49(22.27%)
	N2	51(23.18%)
	N3	74(33.64%)
M stage	M0	194(88.18%)
	M1	26(11.82%)
Disease stage according to TNM	I	31(14.09%)
	II	49(22.27%)
	III	114(51.82%)
	IV	26(11.82%)
Performance status	≤1	178(80.91%)
	>1	42(19.09%)
Surgical status	Pre-operative	69(31.36%)
	Post-operative	151(68.64%)
Surgical treatment	Total gastrectomy	73(48.34%)
	Subtotal gastrectomy	78(51.66%)
Surgical method	Total laparoscopic	32(21.19%)
	Laparoscopic-assisted	94(62.25%)
	Open abdomen	25(16.56%)
Excessive alcohol consumption	Yes	73(33.18%)
	No	147(66.82%)
Smoking status	Smoker	48(21.82%)
	Nonsmoker	172(78.18%)
Weight [kg]	Median (range)	55.0(38.0–95.0)
	<55.0	105(47.73%)
	≥55.0	115(52.27%)
BMI [kg/m2]	Median (range)	20.4(14.2–32.8)
	<20.4	107(48.64%)
	≥20.4	113(51.36%)
NRS-2002	<3	108(49.09%)
	≥3	112(50.91%)
GLIM	No	121(55.00%)
	Yes	99(45.00%)
PG-SGA	<4	78(35.45%)
	≥4	142(64.55%)
TNF-α genotype	CC(homozygous mutation)	7(3.18%)
	CT(heterozygous mutation)	61(27.73%)
	TT(wild type)	152(69.09%)
TNF-α genotype in Malnutrition Patients (GLIM)	CC	6(6.06%)
	CT	35(35.35%)
	TT	58(58.59%)

### 3.2. Impact of demographic and clinical variables on the risk of malnutrition

#### 3.2.1. Univariable analysis

According to the NRS-2002 scale, the risk of a higher degree (≥3) of nutritional risk was increased 2.0-fold in old patients (age ≥ 65 years) (*p* = 0.010; OR = 2.083). And more than 2.9 times higher risk was observed in patients with lymph node involvement (*p* = 0.003; OR = 2.947). In patients with stage III-IV, this risk has risen 2-fold. (*p* = 0.017; OR = 2.000). For patients with poor performance status, the nutritional risk was nearly 10.1 times higher (*p* < 0.001; OR = 10.163). Patients who underwent surgery are at a 2.0-fold higher risk of nutritional risk (*p* = 0.020; OR = 2.003) (Table 2).

**Table 2:** Impact of demographic, clinical and genetic variables on the NRS-2002.

Variable		NRS-2002		
		≥3	<3	Univariable analysis
				OR [95% CI] *p*
Gender	Male	77 (50.00%)	77 (50.00%)	0.886 [0.497–1.578] 0.769
	Female	35 (53.03%)	31 (46.97%)	
Age [years]	≥65	68 (59.65%)	46 (40.35%)	2.083 [1.216–3.587] 0.010*
	<65	44 (41.51%)	62 (58.49%)	
Histopathological diagnosis	Adenocarcinoma	96 (53.33%)	84 (46.67%)	1.714 [0.854–3.442] 0.162
	Non-Adenocarcinoma	16 (40.00%)	24 (60.00%)	
Tumor location	Low third	62 (48.82%)	65 (51.18%)	0.820 [0.480–1.402] 0.497
	Middle and upper third	50 (53.76%)	43 (46.24%)	
T stage	T4	47 (55.95%)	37 (44.05%)	1.388 [0.803–2.397] 0.268
	T1–3	65 (47.79%)	71 (52.21%)	
N stage	N1–3	98 (56.32%)	76 (43.68%)	2.947 [1.470–5.911] 0.003*
	N0	14 (30.43%)	32 (69.57%)	
M stage	M1	15 (57.69%)	11 (42.31%)	0.733 [0.321–1.677] 0.534
	M0	97 (50.00%)	97 (50.00%)	
Disease stage according to TNM	IV	15 (57.69%)	11 (42.31%)	0.733 [0.321–1.677] 0.534
	I–III	97 (50.00%)	97 (50.00%)	
	III–IV	80 (57.14%)	60 (42.86%)	2.000 [1.144–3.497] 0.017*
	I–II	32 (40.00%)	48 (60.00%)	
Performance status	>1	37 (88.10%)	5 (11.90%)	10.163 [3.814–27.081] <0.001*
	≤1	75 (42.13%)	103 (57.87%)	
Excessive alcohol consumption	Yes	37 (50.69%)	36 (49.31%)	0.987 [0.563–1.730] 1.000
	No	75 (51.02%)	72 (48.98%)	
Smoking status	Smoker	22 (45.83%)	26 (54.17%)	0.771 [0.406–1.465] 0.514
	Nonsmoker	90 (52.33%)	82 (47.67%)	
Surgical status	Post-operative	85 (56.29%)	66 (43.71%)	2.003 [1.121–3.580] 0.020*
	Pre-operative	27 (39.13%)	42 (60.86%)	
Surgical treatment	Total gastrectomy	47 (64.38%)	26 (35.62%)	1.903 [0.990–3.656] 0.071
	Subtotal gastrectomy	38 (48.72%)	40 (51.28%)	
Surgical method	Laparoscopic-assisted and open abdomen	68 (57.14%)	51 (42.86%)	1.176 [0.537–2.575] 0.693
	Total laparoscopic	17 (53.12%)	15 (46.88%)	
	Open abdomen	16 (64.00%)	9 (36.00%)	1.469 [0.604–3.572] 0.509
	Total laparoscopic and Laparoscopic-assisted	69 (54.76%)	57 (45.24%)	
TNF-α genotype	CC	6 (85.71%)	1 (14.29%)	6.057 [0.717–51.168] 0.119
	CT and TT	106 (49.77%)	107 (50.23%)	
	TT	69 (45.39%)	83 (54.61%)	0.483 [0.269–0.870] 0.019*
	CC and CT	43 (63.24%)	25 (36.76%)	
	CT	37 (60.66%)	24 (39.34%)	1.727 [0.947–3.148] 0.097
	CC and TT	75 (47.17%)	84 (52.83%)	

*Statistically significant, namely *p* < 0.05.

According to GLIM, for patients ≥65 years, the risk of malnutrition was increased by about a factor of 1.8 (*p* = 0.042; OR = 1.770). The risk of malnutrition was also elevated in patients with lymphatic metastases and III-IV (*p* = 0.001; OR = 3.256 and *p* = 0.003; OR = 2.467 respectively). And an 8.9 times higher risk of malnutrition was observed in the poor performance status patients (*p* < 0.001; OR = 8.906). For post-operative patients, the risk of malnutrition was elevated (*p* = 0.001; OR = 2.688). However, the risk of malnutrition was reduced by a factor of 2.9 in smokers (*p* = 0.034; OR = 0.476) (Table 3).

**Table 3:** Impact of demographic, clinical, nutritional and genetic variables on the GLIM.

Variable		GLIM		
		Yes	No	Univariable analysis
				OR [95% CI] *p*
Gender	Male	67 (43.51%)	87 (56.49%)	0.818 [0.459–1.459] 0.555
	Female	32 (48.48%)	34 (51.52%)	
Age [years]	≥65	59 (51.75%)	55 (48.25%)	1.770 [1.034–3.031] 0.042*
	<65	40 (37.74%)	66 (62.26%)	
Histopathological diagnosis	Adenocarcinoma	85 (47.22%)	95 (52.78%)	1.662 [0.815–3.389] 0.218
	Non-Adenocarcinoma	14 (35.00%)	26 (65.00%)	
Tumor location	Low third	53 (42.06%)	73 (57.94%)	0.789 [0.461–1.351] 0.413
	Middle and Upper third	45 (48.39%)	48 (51.61%)	
T stage	T4	44 (52.38%)	40 (47.62%)	1.620 [0.936–2.803] 0.095
	T1–3	55 (40.44%)	81 (59.56%)	
N stage	N1–3	88 (50.57%)	86 (49.43%)	3.256 [1.554–6.823] 0.001*
	N0	11 (23.91%)	35 (76.09%)	
M stage	M1	13 (50.00%)	13 (50.00%)	0.796 [0.351–1.807] 0.676
	M0	86 (44.33%)	108 (55.67%)	
Disease stage according to TNM	IV	13 (50.00%)	13 (50.00%)	0.796 [0.351–1.807] 0.676
	I–III	86 (44.33%)	108 (55.67%)	
	III–IV	74 (52.86%)	66 (47.14%)	2.467 [1.384–4.395] 0.003*
	I–II	25 (31.25%)	55 (68.75%)	
Performance status	>1	35 (83.33%)	7 (16.67%)	8.906 [3.741–21.202] <0.001*
	≤1	64 (35.96%)	114 (64.04%)	
Excessive alcohol consumption	Yes	30 (41.10%)	43 (58.90%)	0.789 [0.447–1.391] 0.472
	No	69 (46.94%)	78 (53.06%)	
Smoking status	Smoker	15 (31.25%)	33 (68.75%)	0.476 [0.241–0.940] 0.034*
	Nonsmoker	84 (48.84%)	88 (51.16%)	
Surgical Status	Post-operative	79 (56.29%)	72 (43.71%)	2.688 [1.460–4.949] 0.001*
	Pre-operative	20 (39.13%)	49 (60.86%)	
Surgical treatment	Total Gastrectomy	44 (60.27%)	29 (39.73%)	1.864 [0.976–3.561] 0.073
	Subtotal Gastrectomy	35 (44.87%)	43 (55.13%)	
Surgical method	Laparoscopic-assisted and Open abdomen	65 (54.62%)	54 (45.38%)	1.548 [0.705–3.397] 0.321
	Total laparoscopic	14 (43.75%)	18 (56.25%)	
	Open abdomen	15 (60.00%)	10 (40.00%)	1.453 [0.607–3.479] 0.512
	Total laparoscopic and Laparoscopic-assisted	64 (50.79%)	62 (49.21%)	
TNF-α genotype	CC	6 (85.71%)	1 (14.29%)	7.742 [0.916–65.425] 0.048*
	CT and TT	93 (43.66%)	120 (56.34%)	
	TT	58 (38.16%)	94 (61.84%)	0.406 [0.226–0.730] 0.003*
	CT and CC	41 (60.29%)	27 (39.71%)	
	CT	35 (57.38%)	26 (42.62)	1.998 [1.098–3.635] 0.024*
	CC and TT	64 (40.25%)	95 (59.75%)	

*Statistically significant, namely *p* < 0.05.

According to the PG-SGA scale, older patients (≥65 years) are at higher risk for malnutrition (*p* < 0.001; OR = 2.988). A 5.6-fold higher risk of a diagnosis of malnourished status was noted in the case of people with lymph node involvement (N1–N3) (*p* < 0.001; OR = 5.584). The risk of malnutrition was significantly higher in patients with stages III-IV (*p* = 0.001; OR = 2.684). In addition, a significantly higher risk of malnutrition was observed in patients who underwent surgery (*p* = 0.001; OR = 2.831), especially those who underwent total gastrectomy (*p* < 0.001; OR = 3.718) (Table 4).

**Table 4:** Impact of demographic, clinical and genetic variables on the PG-SGA.

Variable		PG-SGA		
		≥4	<4	Univariable analysis
				OR [95% CI] *p*
Gender	Male	105 (68.18%)	49 (31.82%)	1.680 [0.929–3.038] 0.93
	Female	37 (56.06%)	29 (43.94%)	
Age [years]	≥65	87 (76.32%)	27 (23.68%)	2.988 [1.680–5.314] <0.001*
	<65	55 (55.89%)	51 (48.11%)	
Histopathological diagnosis	Adenocarcinoma	120 (66.67%)	60 (33.33%)	1.636 [0.816–3.281] 0.201
	Non-adenocarcinoma	22 (55.00%)	18 (45.00%)	
Tumor location	Low third	81 (63.78%)	46 (36.22%)	0.924 [0.527–1.618] 0.887
	Middle and Upper third	61 (65.59%)	32 (34.41%)	
T stage	T4	57 (67.86%)	27 (32.14%)	1.267 [0.713–2.250] 0.470
	T1–3	85 (62.50%)	51 (37.50%)	
N stage	N1–3	127 (72.99%)	47 (27.01%)	5.584 [2.769–11.262] <0.001*
	N0	15 (32.61%)	31 (67.39%)	
M stage	M1	20 (76.92%)	6 (23.08%)	0.508 [0.195–1.325] 0.194
	M0	122 (62.89%)	72 (37.11)	
Disease stage according to TNM	IV	21 (80.77%)	5 (19.23%)	0.411 [0.159–1.222] 0.125
	I–III	126 (64.95%)	68 (35.05%)	
	III–IV	102 (72.86%)	38 (27.14%)	2.684 [1.510–4.771] 0.001*
	I–II	40 (50.00%)	40 (50.00%)	
Performance status	>1	42 (100.00%)	0 (0.00%)	0.704 [0.633–0.783] <0.001*
	≤1	100 (56.18%)	78 (43.82%)	
Excessive alcohol consumption	Yes	52 (71.23%)	21 (28.77%)	1.568 [0.856–2.874] 0.178
	No	90 (61.22%)	57 (38.78%)	
Smoking status	Smoker	33 (68.75%)	15 (31.25%)	1.272 [0.641–2.522] 0.503
	Nonsmoker	109 (63.37%)	63 (36.63%)	
Surgical Status	Post-operative	109 (72.19%)	42 (27.81%)	2.831 [1.567–5.115] 0.001*
	Pre-operative	33 (47.83%)	36 (52.17%)	
Surgical treatment	Total Gastrectomy	62 (84.93%)	11 (15.07%)	3.718 [1.695–8.154] <0.001*
	Subtotal Gastrectomy	47 (60.26%)	31 (39.74%)	
Surgical method	Laparoscopic-assisted and Open abdomen	88 (73.95%)	31 (26.05%)	1.487 [0.644–3.432] 0.378
	Total laparoscopic	21 (65.62%)	11 (34.38%)	
	Open abdomen	21 (84.00%)	4 (16.00%)	2.2671 [0.729–7.053] 0.221
	Total laparoscopic and Laparoscopic-assisted	88 (69.84%)	38 (30.16%)	
TNF-α genotype	CC	5 (71.43%)	2 (28.57%)	1.387 [0.263–7.320] 1.000
	CT and TT	137 (64.32%)	76 (35.68%)	
	TT	94 (61.84%)	58 (38.16%)	0.675 [0.365–1.250] 0.226
	CC and CT	48 (70.59%)	20 (29.41%)	
	CT	43 (70.49%)	18 (29.51%)	1.448 [0.766–2.737] 0.274
	CC and TT	99 (62.26%)	60 (37.74%)	

*Statistically significant, namely *p* < 0.05.

#### 3.2.2. Multivariable analysis

According to the NRS-2002 scale, independent predictors of higher risk of malnutrition include advanced age (≥65) (*p* = 0.018; OR = 2.106), lymph node involvement (N1–N3) (*p* = 0.041; OR = 2.725) and worse performance status (>1) (*p* < 0.001; OR = 9.073). According to GLIM, independent predictors of higher risk of malnutrition include advanced age (≥65) (*p* = 0.035; OR = 2.028), worse performance status (>1) (*p* < 0.001; OR = 9.540) and surgery (*p* = 0.020; OR = 2.316). But the smoker (*p* = 0.006; OR = 0.301) were found to be significantly related to a lower risk of malnutrition. According to PG-SGA, independent predictors of higher risk of malnutrition include advanced age (≥65) (*p* < 0.001; OR = 3.524), lymph node involvement (N1–N3) (*p* < 0.001; OR = 5.921) and undergoing surgery (*p* = 0.002; OR = 2.886) (Table 5).

**Table 5:** Impact of demographic, clinical, nutritional and genetic variables on the risk of malnutrition.

Variable		NRS-2002	GLIM	PG-SGA
		Multivariable analysis		
		OR [95% CI] *p*		
Age [years]	≥65	2.106 [1.135–3.906] 0.018*	2.028 [1.049–3.922] 0.035*	3.524 [1.847–6.723] <0.001*
	<65			
N stage	N1–3	2.725 [1.044–7.108] 0.041*	2.387 [0.804–7.086] 0.117	5.921 [2.312–15.161] <0.001*
	N0			
Disease stage according to TNM	III–IV	1.086 [0.500–2.359] 0.835	1.615 [0.687–3.793] 0.272	1.106 [0.493–2.481] 0.807
	I–II			
Performance status	>1	9.073 [3.292–25.003] <0.001*	9.540 [3.613–25.192] <0.001*	–
	≤1			
Smoking status	Smoker	–	0.301 [0.127–0.709] 0.006*	–
	Nonsmoker			
Surgical Status	Post-operative	1.599 [0.837–3.055] 0.155	2.316 [1.141–4.704] 0.020*	2.886 [1.482–5.623] 0.002*
	Pre-operative			
TNF-α genotype	CC	–	20.114 [1.889–214.121] 0.013*	–
	CT and TT			
	TT	0.340 [0.174–0.665] 0.002*	–	–
	CC and CT			
	CT	–	3.218 [1.564–6.624] 0.002*	–
	CC and TT			

*Statistically significant, namely *p* < 0.05.

### 3.3. Impact of genetic variables on the risk of malnutrition

#### 3.3.1. Univariable analysis

According to the NRS-2002 scale, the presence of the TT genotype reduced (approximately 2.1-fold) the risk of malnutrition (*p* = 0.019; OR = 0.483) (Table 2). According to GLIM, patients carrying the C allele were at increased risk of malnutrition compared to the TT genotype (*p* = 0.003; OR = 0.406). Patients with the CT genotype had a 2.0-fold increased risk of malnutrition (*p* = 0.024; OR = 1.998), while those with the CC genotype had a 7.7-fold increased risk of malnutrition (*p* = 0.048; OR = 7.742) (Table 3).

#### 3.3.2. Multivariable Analysis

According to the NRS-2002 scale, the TT genotype is a protective factor for malnutrition (*p* = 0.002; OR = 0.340). According to GLIM, independent predictors of higher risk of malnutrition include CC (*p* = 0.013; OR = 20.114) and CT (*p* = 0.002; OR = 3.218) genotype of the TNF-α gene (Table 5).

## 4. Discussion

Malnutrition can occur in patients with early-stage cancer, but is more prevalent in patients with advanced cancer and can progress to cancerous cachexia (17, 18). According to statistics, about 80% of patients with upper gastrointestinal tract tumors can develop cancerous cachexia (40). It seriously affects the quality of life of patients with tumors, shortens their survival, affects the implementation of treatment plans, reduces the sensitivity of treatment, increases the occurrence of complications, and is an important cause of death in patients with tumors (10). With continued research into the molecular mechanisms of cancerous cachexia, it is now believed that the systemic inflammatory response plays a role in the progression of cancer and cancer-related cachexia. Inflammation is mediated by a network of pro- and anti-inflammatory cytokines that are normally in homeostasis. In the cancer state, homeostasis is disrupted, leading to a dysfunctional state of both immune stimulation and suppression (41). Studies have shown that the role of cytokines in cellular events determines the onset, promotion, invasion, and metastasis of cancer (42). Similarly, Fearon et al. have highlighted the production of several cytokines associated with the prevalence of cachexia in a variety of cancers (20). Although cytokine levels have been associated with cancer and cachexia in numerous studies, the mechanisms by which these substances act on tumors and other body systems are not fully understood. Ghrelin, a peptide hormone secreted mainly by the stomach, plays a crucial role in eliminating hunger and maintaining a state of energy homeostasis and has been shown to increase appetite and calorie intake in both healthy individuals and cancerous cachexia. Appetite and calorie intake in healthy individuals and animal models of cancerous cachexia (43). Gastrin levels have been reported to be 50% higher in cancer patients with cachexia (44). These elevated gastrin levels may represent a counter-regulatory mechanism against anorexia associated with tumour growth. Studies have also shown that leptin levels are positively correlated with body fat mass and that any dynamic change in plasma leptin concentration activates energy regulation pathways (45, 46). Leptin can reduce food intake and increase energy expenditure via hypothalamic neuropeptides downstream of leptin. In the tumour-carrying state, malnutrition factors, such as cytokines, can mimic the excessive transmission of negative feedback signals from leptin, thereby inducing anorexia and weight loss, and ultimately malnutrition. However, there is still no effective treatment for cancer malnutrition. Early identification of patients at risk of malnutrition and timely and accurate nutritional interventions can therefore prevent the onset of malnutrition or progression of malnutrition to cancer cachexia (47). In this study, by testing serum TNF-α gene polymorphism (-1031T/C, rs1799964) in GC patients, it was found that the development of malnutrition in gastric cancer was associated with serum TNF-α gene polymorphism.

Currently, some TNF-α SNPs have been studied. Murdaca et al. found that the TNF-α-489 variant allele A was significantly associated with susceptibility to psoriatic arthritis and with the severity of clinical and laboratory parameters (35). In addition, both SNPs +489 GG and + 489 GA genotypes responded to etanercept for psoriatic arthritis, whereas no response was observed in patients carrying the SNP +489 AA genotype (48–50). Studies have shown that sHLA-G produced by lamina propria monocytes may induce high TNF-α expression and aggravate tissue damage (51). Whereas more frequent HLA-G expression was observed in patients with advanced stages of gastric cancer, lymph node involvement, or advanced T-stage, affecting the prognosis of gastric cancer patients, which may be related to the induction of high TNF-α expression by HLA-G (52). The body is in a state of stress during malignancy and produces large amounts of TNF-α, which acts on various organs, suppressing the patient’s appetite and affecting the body’s protein, sugar and fat metabolism (53–56). TNF-α can act directly on the hypothalamus to produce an anorexic response in the body. Studies have shown that rats transfected with the human TNF-α gene exhibit anorexia nervosa (57). TNF-α causes oxidative stress and nitric oxide synthesis, leading to a decrease in myocardin and creatine phosphokinase activity, resulting in skeletal muscle wasting (58). TNF-α also directly activates muscle proteolysis, accelerating changes in protein metabolism and leading to altered plasma amino acid profiles, with increased plasma tryptophan concentrations playing a key role in progressive nutrient depletion in malnourished patients. TNF-α can lead to increased gluconeogenesis and impaired glucose utilization in peripheral tissues, and can also induce insulin resistance in tumour patients (59). TNF-a can inhibit the lipogenic activity of several lipases, including LPL, inhibit lipid synthesis and reduce LPL mRNA expression, while leading to hyperlipidaemia. TNF-α can also upregulate the expression of uncoupling protein (UCP) 2 and 3 in skeletal muscle, promoting thermogenesis and thereby accelerating skeletal muscle catabolism (60). TNF-α has also been associated with DNA fragmentation and myocyte apoptosis in skeletal muscle cells in a cancerous cachectic state (61). Murray et al. infected severe combined immunodeficient mice with two tumour strains (Morris 7777 hepatocellular carcinoma and MCA sarcoma), resulting in cachexia with a significant increase in TNF-α levels in the former and no expression of tumour growth cytokines in the latter (62). In C-26-induced cancer cachexia mice, pre-administration of adalimumab (a TNF-α inhibitor) significantly reduced cancer cachexia, as evidenced by significantly reduced weight loss, and leg muscle retention (63). These demonstrate esethat TNF-α plays a key role in cancer cachexia. Several polymorphisms of TNF-α have also been described in the available literature as detrimental factors contributing to malnutrition. Sharma et al. found that TNF-α -238 AA and -308 AA genotype in patients with end-stage renal disease exhibited a higher susceptibility to malnutrition and higher levels of TNF-α (31). Homa-Mlak et al. investigated the association between single nucleotide polymorphisms in the TNF-α-610 T > G (rs4149570) gene and the development of malnutrition in HNC patients receiving radiotherapy (23). They found that the TT genotype possessed a higher risk of malnutrition (OR = 5.05; *p* = 0.0350) and was also associated with critical weight loss (OR = 24.85; *p* = 0.0009). The association of TNF-α-1031T/C SNP with malnutrition or inflammation has also been partially investigated. Hernández-Díaz et al. identified TNF-α-1031T/C SNP as a possible risk factor for coronary heart disease through a meta-analysis of inflammatory markers of cardiovascular heart disease (64). Negoro and Sanchez et al. found a high prevalence of the TNF-α-1031 CC genotype in patients with Crohn’s disease by individuals with ulcerative colitis compared to healthy controls (65, 66). A recent study by Nourian et al. also suggests that the TNF-α-1031 CC genotype may be associated with genetic risk of inflammatory bowel disease (67). Furthermore, TNF-α mRNA expression was significantly higher in the CC genotype carrier group compared to CT or TT genotype carriers. Johns et al. found a positive association between TNF-α-1031T/C and weight loss >2% and low skeletal muscle index in a study of approximately 1200 individuals with weight loss and muscle wasting (*p* = 0.010) (33). Powrózek et al. suggested for the first time that the TNF-α-1031T/C (rs1799964) polymorphism might be associated with the development of cachexia through a study of 62 HNC patients undergoing RT (25). The authors found that the CC genotype was considered to have a higher risk of cachexia (*p* = 0.044; HR = 3.724) and that patients with the CC genotype had significantly lower body weight (*p* = 0.045) and higher TNF-α plasma levels (*p* = 0.006). In addition, the authors found that CC genotype carriers had a significantly higher risk of early mortality (HR = 3.630, *p* = 0.013). Yehia et al. found that TNF-α polymorphisms (rs1800629; −308 G/A) were positively associated with pancreatic and non-small cell lung cancer cohorts through a study of pancreatic and non-small cell lung cancer cohorts; conversely, (rs1799964; -1031T/C) may act as a protective factor against cachexia in patients with non-small cell lung cancer (34).

In our study, we assessed the impact of demographic, clinical and genetic variables on malnutrition in GC patients by PG-SGA score, NRS-2002 score and GLIM clinical diagnosis of malnutrition. We found that patients carrying the C allele, especially carrying the CC genotype, were at increased risk of malnutrition. However, our study still has some limitations. First, we did not assess the impact of diet or food intake problems due to the presence of the tumor itself on the development of nutritional disorders. In addition, we did not assess the effect of the individual genotype of the studied gene on its expression (and the expression of the protein it encodes). Despite these limitations, TNF-α -1,031 T/C gene polymorphism is still a potential predictor of GC malnutrition. Further studies need to be conducted. Perhaps in the future, patients carrying the unfavorable C allele could be scheduled for earlier pharmacological intervention to prevent them from developing severe malnutrition or cachexia.

## Data availability statement

The original contributions presented in the study are included in the article/supplementary materials, further inquiries can be directed to the corresponding authors.

## Ethics statement

This study was reviewed and approved by the Medical Ethics Committee of Jinhua Municipal Central Hospital [(Research) 2020-Ethical Review-240, (Research) 2021-Ethical Review-16 and (Research) 2021-Ethical Review-16]. The patients/participants provided their written informed consent to participate in this study.

## Author contributions

LF developed the conception and design of this study. LF, YC, RZ, MZ, and LD collected the primary data. LF, CL, LZ, DG, and XY made contributions to analysis and interpretation of data. LF and CL drafted the manuscript. LZ, DG, and XY revised the manuscript. All authors have given final approval of the version to be published.

## Funding

This study was supported by the General Project of Zhejiang Province Medical Science and Technology Plan (2021KY1181), the Foundation of the Science and Technology Department of Jinhua (2021-3-066), and Jinhua Municipal Central Hospital Foundation (JY2020-5-03).

## Conflict of interest

The authors declare that the research was conducted in the absence of any commercial or financial relationships that could be construed as a potential conflict of interest.

## Publisher’s note

All claims expressed in this article are solely those of the authors and do not necessarily represent those of their affiliated organizations, or those of the publisher, the editors and the reviewers. Any product that may be evaluated in this article, or claim that may be made by its manufacturer, is not guaranteed or endorsed by the publisher.
